
JOURNAL INFORMATION
==============================
NLM Title Abbreviation: Sci China Life Sci
Journal ID: Sci China Life Sci
NLM Journal ID: 101529880

Science China. Life Sciences

ISSN: 1674-7305
EISSN: 1869-1889

ARTICLE INFORMATION
==============================
PMCID: PMC7089182
PMID: 27502905
DOI: 10.1007/s11427-015-0369-x
Article ID: 369
Article version: 1
Subjects: Letter to the Editor

Prevalence and phylogenetic characterization of canine coronavirus from diseased pet dogs in Beijing, China

Lu Shuai 1 2
Zhang Di 3
Zhou Jianfang 2
Xia Zhaofei 3
Lin Degui 3
Lou Yongliang 1
Tan Wenjie tanwj28@163.com
1 2
Qin Kun qkv0115@163.com
2
1 grid.268099.c 0000000103483990 School of Laboratory Medicine and Life Science, Institute of Medical Virology, Wenzhou Medical University, Wenzhou, 325035 China
2 grid.198530.6 0000000088032373 Key Laboratory of Medical Virology, National Health and Family Planning Commission, National Institute for Viral Disease Control and Prevention, Chinese Center for Disease Control and Prevention, Beijing, 102206 China
3 grid.22935.3f 0000000405308290 Department of Small Animal Clinical Sciences, College of Veterinary Medicine, China Agricultural University, Beijing, 100193 China
Electronic publication date: 2016 May 12
Print publication date: 2016
Volume: 59
Issue: 8
First page: 860
Last page: 862
Received 2015 Dec 18; Accepted 2016 Feb 16
Copyright: © The Author(s) 2016
License: Open AccessThis article is distributed under the terms of the Creative Commons Attribution 4.0 International License (https://creativecommons.org/licenses/by/4.0), which permits use, duplication, adaptation, distribution, and reproduction in any medium or format, as long as you give appropriate credit to the original author(s) and the source, provide a link to the Creative Commons license, and indicate if changes were made.
License URL: https://creativecommons.org/licenses/by/4.0/

issue-copyright-statement: © Science in China Press and Springer-Verlag GmbH 2016

==============================

Shuai Lu[1,2†], Di Zhang[3†], Jianfang Zhou[2], Zhaofei Xia[3], Degui Lin[3], Yongliang Lou[1], Wenjie Tan[1,2*] & Kun Qin[2*]

Dear Editor,

Canine coronavirus including canine coronavirus (CCoV) and canine respiratory coronavirus (CRCoV) have been recognized as pathogenic viruses to dogs worldwide, causing enteric and respiratory issues (Buonavoglia et al., 2006). Zoonotic transfer of the viruses from the animal kingdom to humans has been repeatedly observed over the past decade, similarly to SARS- and MERS-coronavirus, which caused a pandemic in 2003 in China and in recent years in the Middle East, respectively (Azhar et al., 2014; Bolles et al., 2011). Thus, studies of the viral prevalence and interspecies transmissions at the animal-human interface are critically important to prepare for possible human pandemics.

In this study, a total of 246 swab samples were collected from 246 corresponding diseased pet dogs visiting the veterinary teaching hospital affiliated with the college of veterinary medicine, China Agricultural University, Beijing. Total RNA were extracted from the 246 sample swabs and subjected to screening for CCoV and CRCoV as well as canine distemper virus (CDV) and canine parainfluenza virus (CPIV) by using a reverse transcription PCR assay targeting the N gene. All PCR amplification products were purified and sequenced to analyze phylogenetic relationships (Table S1 and S2). Data were analyzed by the chi-squared test using SAS software version 9.2 (SAS Institute, Inc., USA). $P$<0.05 was considered statistically significant.

Infections with CCoV were detected in 64 of the 246 surveyed samples, exhibiting the highest infection rate of 26.0% (95%CI: 19.3%–31.9%), followed by 40 for CDV (16.3%, 95%CI: 11.2%–21.3%), 16 for CRCoV (6.5%, 95%CI: 3.3%–9.7%), and 9 for CPIV (3.7% 95%CI: 1.3%–6.0%). Pets aged from several days to many years were infected with the indicated viruses, while 75.2% of the infections mainly occurred in puppies less than 12 months old. According to medical records, the ages of CCoV- and CRCoV-infected dogs were generally less than three months ($P$<0.05), while there was no difference in CDV and CPIV infections. No significant differences were observed regarding gender (Table S3). Notably, it was clear that cases infected with CCoV predominately manifested as gastrointestinal signs, while CDV infections exhibited respiratory symptoms ($P$<0.05). CCoV was predominately examined from rectal swabs, while CDV- and CPIV-positive specimens were mainly found in respiratory tracts ($P$<0.05) (Table S3). Co-infections were also discovered in a single case, CCoV and CDV co-infections were found in nine cases, and four dogs showed CCoV

†Contributed equally to this work
*Corresponding author (email: tanwj28@163.com; qkv0115@163.com)

and CRCoV co-infection, while other co-infection patterns were rarely observed.

Nucleotide sequences in this study were deposited in the Genbank (http://www.ncbi.nlm.nih.gov/genbank) under the accession NO. KR265176–KR265207. Phylogenetic analysis was performed using a portion of N gene partial sequences derived from the positive samples. As coronavirus has been shown to be capable of crossing the species barrier to infect human and cause pandemics, we determined the nearly full-length sequences of the N gene of canine coronavirus. Notably, the N gene sequences of the coronavirus were highly divergent. Phylogenetically, these sequences were divided into three distinct groups. Group 1 included two clear clusters: strains CCoV/BJ-84, 117, 147, 151,161, 171, 174, 176, and 184 grouped closely with the strain detected from the Chinese ferret-badger (*Melogale moschata*) in the wet market and other CCoVs from North America, while CCoV/BJ-87 and 145 fell into another cluster and grouped together with European isolates such as CCoV/23-03/Italy/2004. The N gene of CRCoV/BJ-34, 202, and 221 in group 2 was very similar to that of a South Korea isolate, which also clustered with HCoV-OC43 (Figure 1).

Even the gentlest of our friendly pets can harbor harmful pathogens to both animals and humans. It is necessary to closely evaluate the zoonotic pathogens transferred to other animal hosts or human populations; thus, the best way to prevent and control potential zoonotic infectious diseases is to understand the ecology and evolution of these viruses. Our study examined the prevalence of CCoV and CRCoV as well as the phylogenetic characterization. CCoV showed the highest detection rate among the four viral pathogens, which different from previously reported rates (Erles et al., 2004; Wang et al., 2016). This may be because of the viral spatiotemporal distribution and varied vaccination protocols among regions. Given the complex ecology of viral diseases in pet dogs, many pathogens such as canine parvovirus (CPV), canine adenovirus (CAV), and other common infectious agents were not examined. Thus, co-infections were rarely observed among these investigated viruses. As a nation-wide compulsory vaccination program against coronavirus has not been implemented, the different immunization status of companion animals may select genetic or antigenic variants, leading to the emergence of novel viral strains. Because the N gene was highly conserved among the coronavirus family, segregation of the N gene in the phylogenetic tree implied the genetic diversity of CCoV in this region. Such an ecological niche may provide an optimal opportunity to generate novel viruses with the potential for interspecies transmission, similarly to the emergence of SARS-CoV and MERS-CoV originating in the animal population. However, with increases in the Chinese pet dog population and increasing contact with human population in recent years, new ecology of viral pathogens in canines will

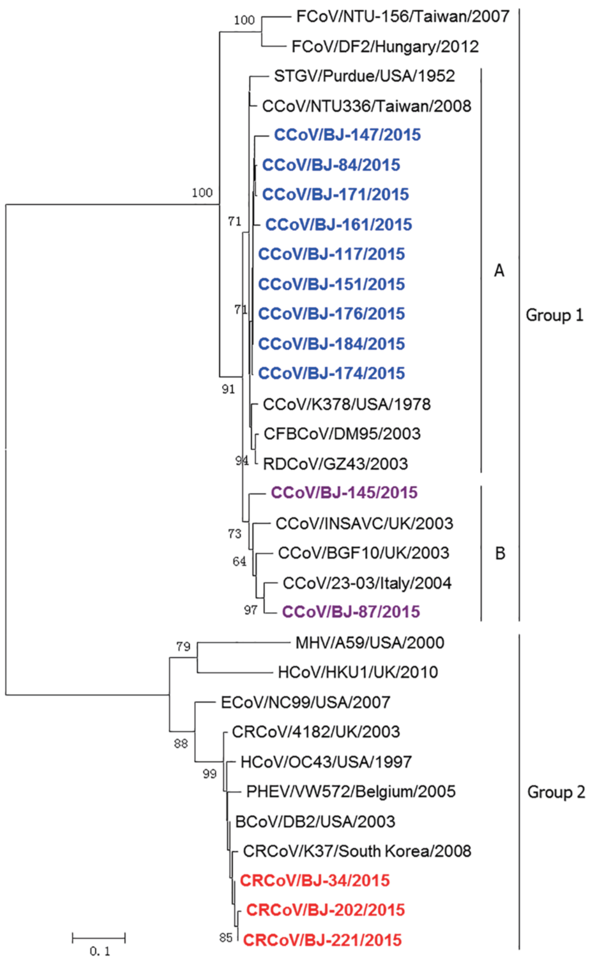

**Figure 1** Phylogenetic relationships between N gene sequence of CCoV and CRCoV. The tree was generated by the neighbor-joining method using the MEGA6 program. Values above the branches are neighbor-joining bootstrap values from 1,000 replicates. Analysis was based on the nearly full-length N gene. Scale bar, 0.1 nucleotide substitution per site. Abbreviations: CFBCoV, Chinese ferret badger coronavirus; RDCoV, raccoon dog coronavirus; STGV, swine transmissible gastroenteritis virus; MHV, murine hepatitis virus; ECoV, equine coronavirus; PHEV, porcine hemagglutinating encephalomyelitis virus; BCoV, bovine coronavirus.

be more complex and elusive. Therefore, it is necessary to strengthen comprehensive surveillance in companion animals in order to prepare for emerging and re-emerging diseases.

**Compliance and ethics** *The author(s) declare that they have no conflict of interest.*

**Acknowledgements** *This work was supported by the State Megaproject for Infectious Disease Research of China (2014ZX10004001-002, 2013ZX10004101, 2013ZX10004601, 2014ZX10004002-001-004 ) and the Teachers' Seed Fund for Research (2014XJ008).*

**Open Access** This article is distributed under the terms of the Creative Commons Attribution License, which permits any use, distribution, and reproduction in any medium, provided the original author(s) and source are credited.

## SUPPORTING INFORMATION

**Table S1** Primers used in this study

**Table S2** Strains of coronavirus used for phylogenetic analysis

**Table S3** Demographic and epidemiological data in this study

The supporting information is available online at life.scichina.com and link.springer.com. The supporting materials are published as submitted, without typesetting or editing. The responsibility for scientific accuracy and content remains entirely with the authors.

Contributed equally to this work

This article is published with open access at link.springer.com


References

Azhar E.I. El-Kafrawy S.A. Farraj S.A. Hassan A.M. Al-Saeed M.S. Hashem A.M. Madani T.A. Evidence for camel-to-human transmission of MERS coronavirus N Engl J Med 2014 370 2499 2505 10.1056/NEJMoa1401505 24896817
Bolles M. Donaldson E. Baric R. SARS-CoV and emergent coronaviruses: viral determinants of interspecies transmission Curr Opin Virol 2011 1 624 634 10.1016/j.coviro.2011.10.012 22180768 PMC3237677
Buonavoglia C. Decaro N. Martella V. Elia G. Campolo M. Desario C. Castagnaro M. Tempesta M. Canine coronavirus highly pathogenic for dogs Emerg Infect Dis 2006 12 492 494 10.3201/eid1203.050839 16704791 PMC3291441
Erles K. Dubovi E.J. Brooks H.W. Brownlie J. Longitudinal study of viruses associated with canine infectious respiratory disease J Clin Microbiol 2004 42 4524 4529 10.1128/JCM.42.10.4524-4529.2004 15472304 PMC522361
Wang X.Y. Li C.Q. Guo D.H. Wang X.Y. Wei S. Geng Y.F. Wang E.Y. Wang Z.H. Zhao X.W. Su M.J. Liu Q.J. Zhang S.Y. Feng L. Sun D.B. Co-circulation of canine coronavirus I and IIa/b with high prevalence and genetic diversity in Heilongjiang province, Northeast China PLoS One 2016 11 0146975 10.1371/journal.pone.0146975 PMC4714894 26771312
